# Supplementary figures and images for: Organelle Genomes of Nardostachys jatamansi Offer New Perspectives into the Evolutionary Dynamics of Caprifoliaceae
Source: Biology (Basel). 2025 Sep 8;14(9):1219. doi: 10.3390/biology14091219 (PMC12467133; doi:10.3390/biology14091219)

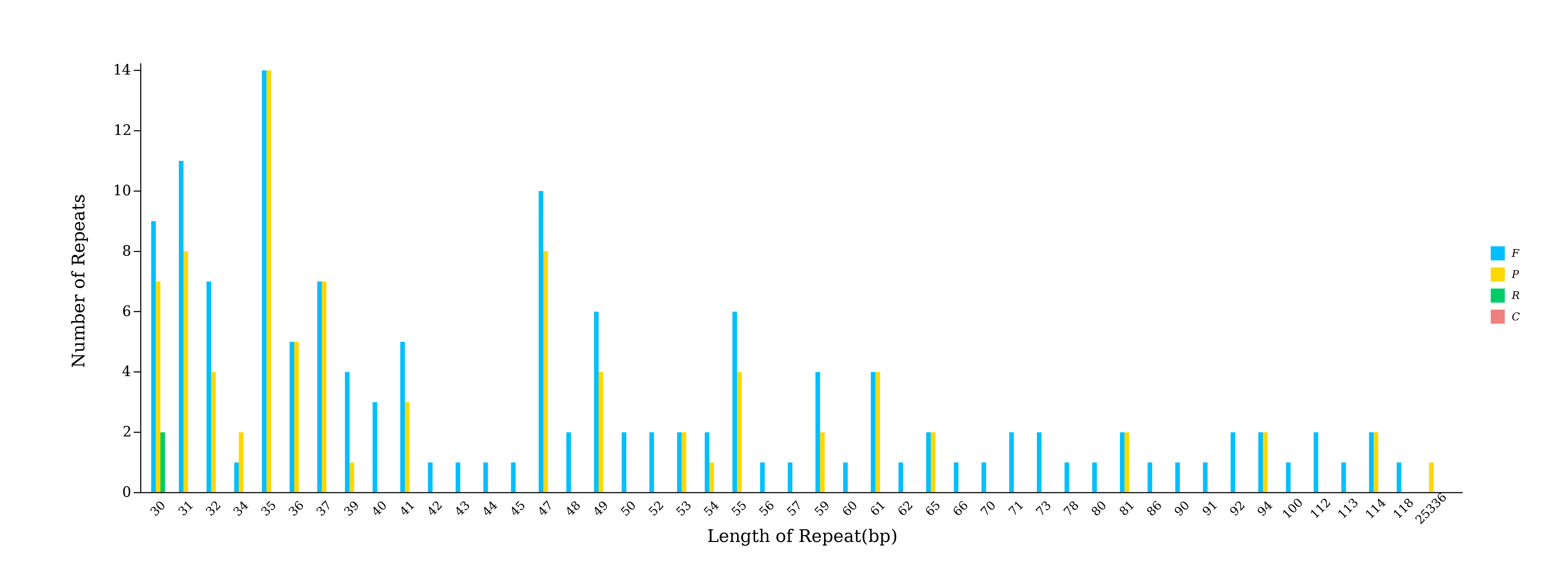

Supplement: Supplementary file 1 [file biology-14-01219-s001.zip › Figure S1.png]

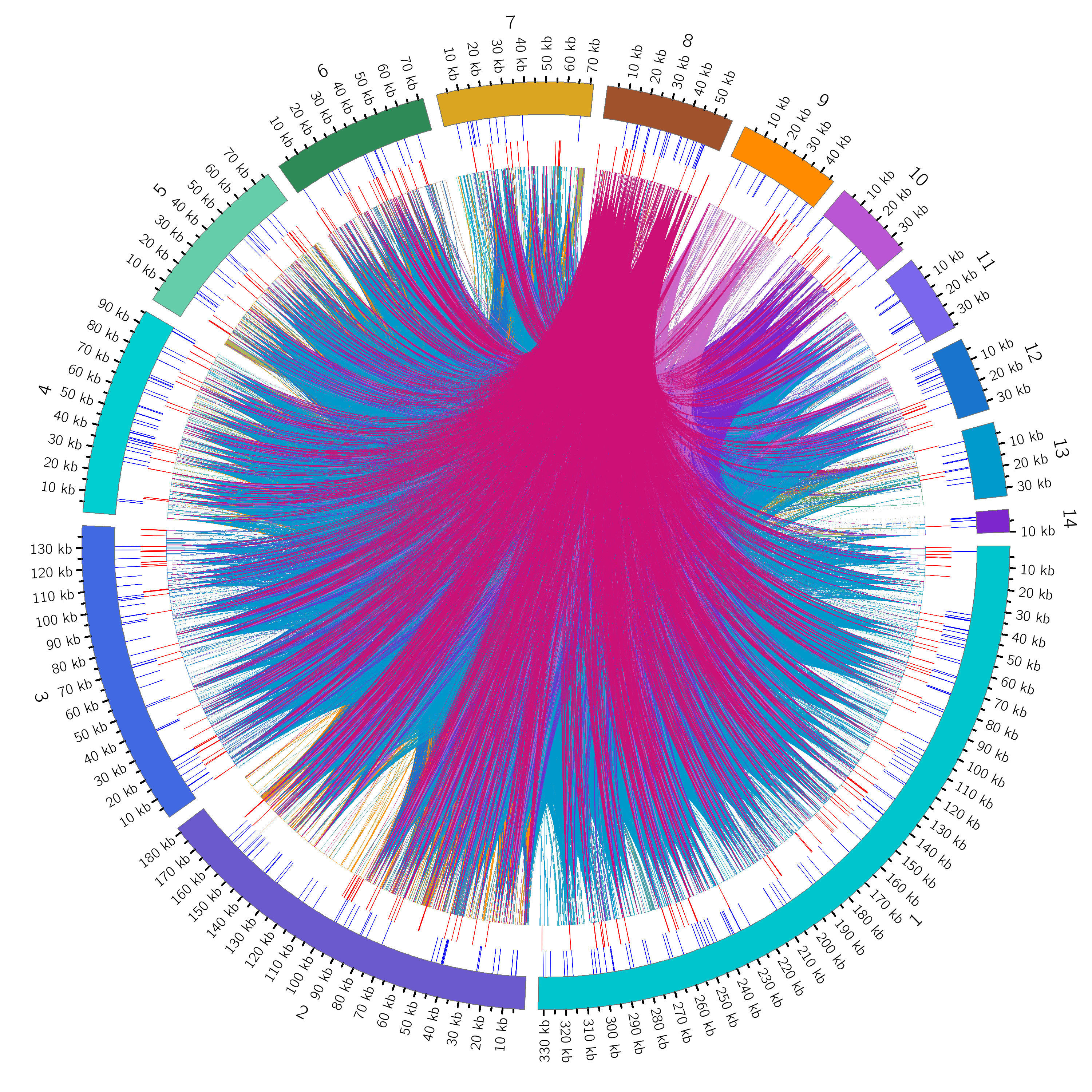

Supplement: Supplementary file 1 [file biology-14-01219-s001.zip › Figure S2.png]

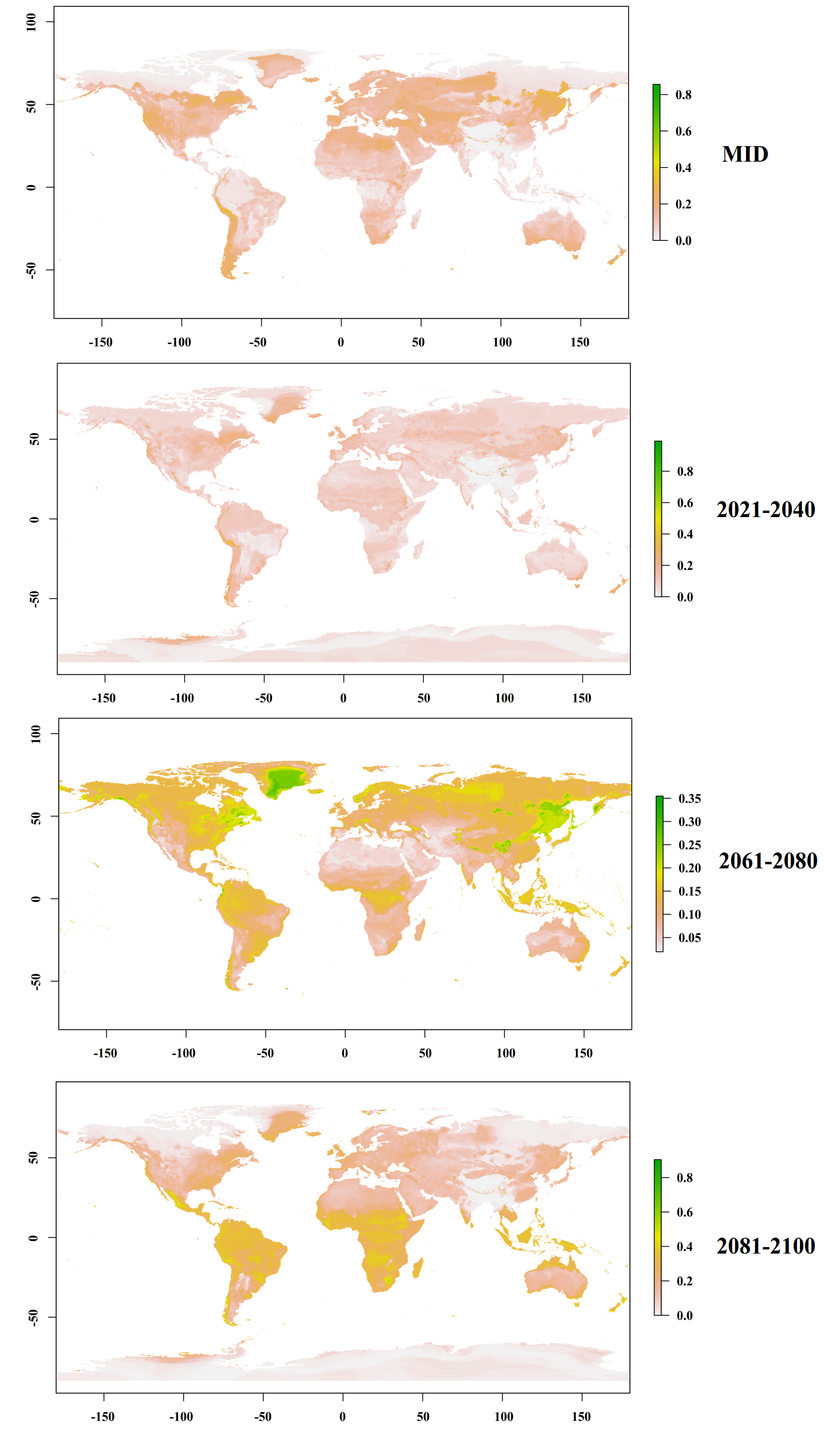

Supplement: Supplementary file 1 [file biology-14-01219-s001.zip › Figure S3.png]
